# Epithelial-mesenchymal transition sensitizes breast cancer cells to cell death via the fungus-derived sesterterpenoid ophiobolin A

**DOI:** 10.1038/s41598-021-89923-9

**Published:** 2021-05-20

**Authors:** Keighley N. Reisenauer, Yongfeng Tao, Provas Das, Shuxuan Song, Haleigh Svatek, Saawan D. Patel, Sheridan Mikhail, Alec Ingros, Peter Sheesley, Marco Masi, Angela Boari, Antonio Evidente, Alexander Kornienko, Daniel Romo, Joseph Taube

**Affiliations:** 1 Department of Biology, Baylor University, Waco, TX USA; 2 Department of Chemistry and Biochemistry, Baylor University, Waco, TX USA; 3 Department of Chemical Sciences, University of Naples Federico II, Complesso Universitario Monte Sant’Angelo, Naples, Italy; 4 Institute of Sciences and Food Production, CNR, Bari, Italy; 5 Department of Chemistry and Biochemistry, Texas State University, San Marcos, TX USA

**Keywords:** Cancer stem cells, Cancer therapy, Cancer, Cell biology

## Abstract

The epithelial–mesenchymal transition (EMT) imparts properties of cancer stem-like cells, including resistance to frequently used chemotherapies, necessitating the identification of molecules that induce cell death specifically in stem-like cells with EMT properties. Herein, we demonstrate that breast cancer cells enriched for EMT features are more sensitive to cytotoxicity induced by ophiobolin A (OpA), a sesterterpenoid natural product. Using a model of experimentally induced EMT in human mammary epithelial (HMLE) cells, we show that EMT is both necessary and sufficient for OpA sensitivity. Moreover prolonged, sub-cytotoxic exposure to OpA is sufficient to suppress EMT-imparted CSC features including sphere formation and resistance to doxorubicin. In vivo growth of CSC-rich mammary cell tumors, is suppressed by OpA treatment. These data identify a driver of EMT-driven cytotoxicity with significant potential for use either in combination with standard chemotherapy or for tumors enriched for EMT features.

## Introduction

Breast cancer patients who have triple-negative breast cancer (TNBC) face poor prognoses driven by high rates of metastasis and early recurrence^1–6^. TNBC is characterized as histologically negative for estrogen receptor (ER), progesterone receptor (PR), and amplified human epidermal growth factor receptor-2 (HER2), preventing the use of hormone- or HER2-targeted therapies. Instead, treatment with anthracycline (doxorubicin) and/or taxanes is capable of providing 5-year survival in only about half of TNBC patients^7–10^.

TNBC is comprised of mostly basal-like and claudin-low intrinsic subtypes, both of which have been characterized as enriched for cancer stem-like cells^11–13^. Cancer stem-like cells (CSCs) are defined by their ability to re-initiate tumor growth upon transplantation and are hypothesized to fuel metastasis and primary tumor recurrence, resulting in an overall decrease in survival^14–17^. To improve TNBC patient outcomes, novel and specific approaches targeted at CSCs are needed.

One proposed mechanism driving the emergence of CSC-like cells is the epithelial-mesenchymal transition (EMT)^16,18^. EMT is a trans-differentiation process characterized by acquisition of a spindle-like morphology, loss of apical-basal polarity, increased motility, and a tolerance to anoikis. These phenotypic shifts are driven by gene expression changes mediated by transcription factors SNAIL (*SNAI1*), TWIST (*TWIST1*), and ZEB1, effects of which include upregulation of vimentin and downregulation of epithelial markers E-cadherin (*CDH1*) and miR-200c^19–25^.

Cells that have undergone an EMT typically acquire CSC properties including decreased sensitivity to conventional chemotherapies used to treat TNBC. This chemoresistance is driven by drug efflux pumps, enhanced DNA repair capacity, and epigenetic changes^16,26–31^. There are currently no approved therapies that specifically target CSCs. A leading pre-clinical compound is salinomycin, reported to decrease the sub-population of CSCs, tumor initiating capability, and chemoresistance, with negligible side effects^32^. Other naturally occurring compounds such as curcumin and quercetin have been reported to reduce the effects of EMT by inhibiting key proteins associated with migration (SNAIL, MMP-2/9), anoikis tolerance (BCL2), cell-to-cell adhesion (N-cadherin), and signaling cascades (JAK/STAT, ERK)^33–36^. This breadth illustrates the potential for applying natural products to persistent issues in oncology.

Ophiobolin A (OpA) is a natural product produced by fungi in the genera *Aspergillus, Bipolaris, Cephalosporium, Cochliobolus,* and *Drechslera*^37^. This sesterterpenoid (25-carbons) is a secondary metabolite that has long been studied for its phytotoxic effects in a variety of plants and has begun to be evaluated for its anti-cancer properties ^37^. Published cell culture-based experiments describe a role for OpA in motility inhibition^38^, membrane depolarization^39–42^, roles in inflammation^43^, and reduction in stemness^44^. In vivo data demonstrate that OpA is tolerated in mice and is effective in an orthotopic model of glioblastoma^39,45,46^. Herein, we investigated the applicability of OpA on EMT-enriched breast cancer and found that experimentally induced EMT enhances the susceptibility of mammary epithelial cells to OpA-induced cell death. Furthermore, breast cancer cell lines treated with OpA experience loss of EMT-associated stemness attributes, demonstrating that OpA induces selective cytotoxicity in cells that have undergone EMT. Additionally, OpA is effective in reducing tumor burden in mice with orthotopic, EMT-positive, mammary tumors, highlighting the potential of EMT-targeted cancer treatment.

## Results

### Mammary epithelial cells that have undergone EMT are more sensitive to OpA

**Figure 1:**
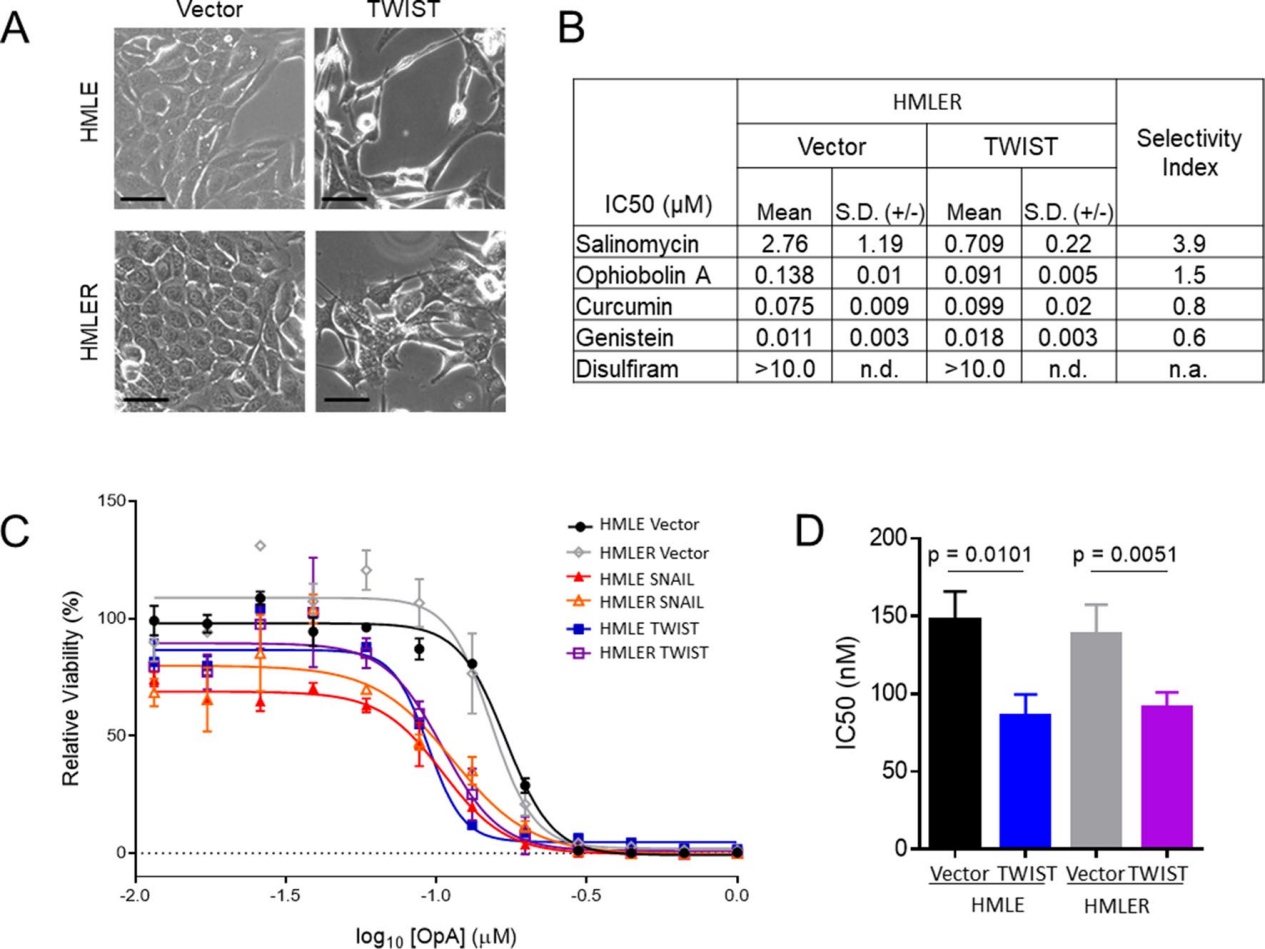
Sensitivity to OpA is enhanced by EMT. (**A**) Representative morphology of non-transformed, immortalized, mammary epithelial cells expressing TWIST or control vector. Scale bar represents 20 µm. (**B**) Cytotoxic activity of the indicated compounds was measured, in triplicate, by MTS assay. Mean and standard deviation of IC_50_ values are reported. Selectivity index is calculated as (HMLER Vector IC_50_))/(HMLER TWIST IC_50_). (**C**) Representative data indicating cytotoxicity for the indicated cell lines. Error bars represent standard deviation. (**D**) Mean and standard deviation of IC_50_ values for OpA, n = 3 or 4, two-tailed student's unpaired t-test used to test significance. n.d. = not determined, n.a. = not applicable.

Given the previously published link between OpA and CSC-targeted activity^44^, we investigated a potential link between OpA and EMT using an experimental model of EMT induction. Immortalized human mammary epithelial (HMLE) cells have an epithelial morphology (Fig. 1A) and express E-cadherin (Sup Fig. 1A). We used HMLE cells, as well as HMLE cells transformed with the Ras oncoprotein (HMLER) that are induced to undergo EMT through lentiviral transduction of the EMT-inducing transcription factor (TF) TWIST^25,47^, resulting in the acquisition of a mesenchymal morphology (Fig. 1A) and protein expression (Sup Fig. 1A). TWIST expression also induces stemness properties including a greater prevalence of cells expressing high levels of CD44 and low levels of CD24 (CD44^hi^/CD24^lo^) (Sup Fig. 1B) and an increased sphere formation efficiency (Sup Fig. 1C)^16,25^. To identify EMT-selective, highly active molecules, we measured the level of TWIST-induced sensitivity to molecules shown to inhibit CSC properties including salinomycin^48^, OpA^44^, curcumin^49^, genistein^50^, and disulfiram^51^. Only two such molecules demonstrated selectivity towards EMT-positive cells, salinomycin and OpA, and only OpA also demonstrated consistently sub-micromolar cytotoxic activity (Fig. 1B). Furthermore, induction of EMT through expression of TWIST or through another EMT-TF, SNAIL^52^, in either HMLE or HMLER cells increased sensitivity to OpA-driven cytotoxicity (Fig. 1C). Indeed, EMT decreased the IC_50_ value from a mean of 137–147 nM for epithelial cells to a mean of 85–91 nM for mesenchymal cells (Fig. 1D). These results stand in stark contrast to EMT-driven resistance to many commonly used chemotherapeutic drugs including doxorubicin and staurosporine (Sup Fig. 1D).

### miR-200c suppression is necessary for sensitivity to OpA

**Figure 2:**
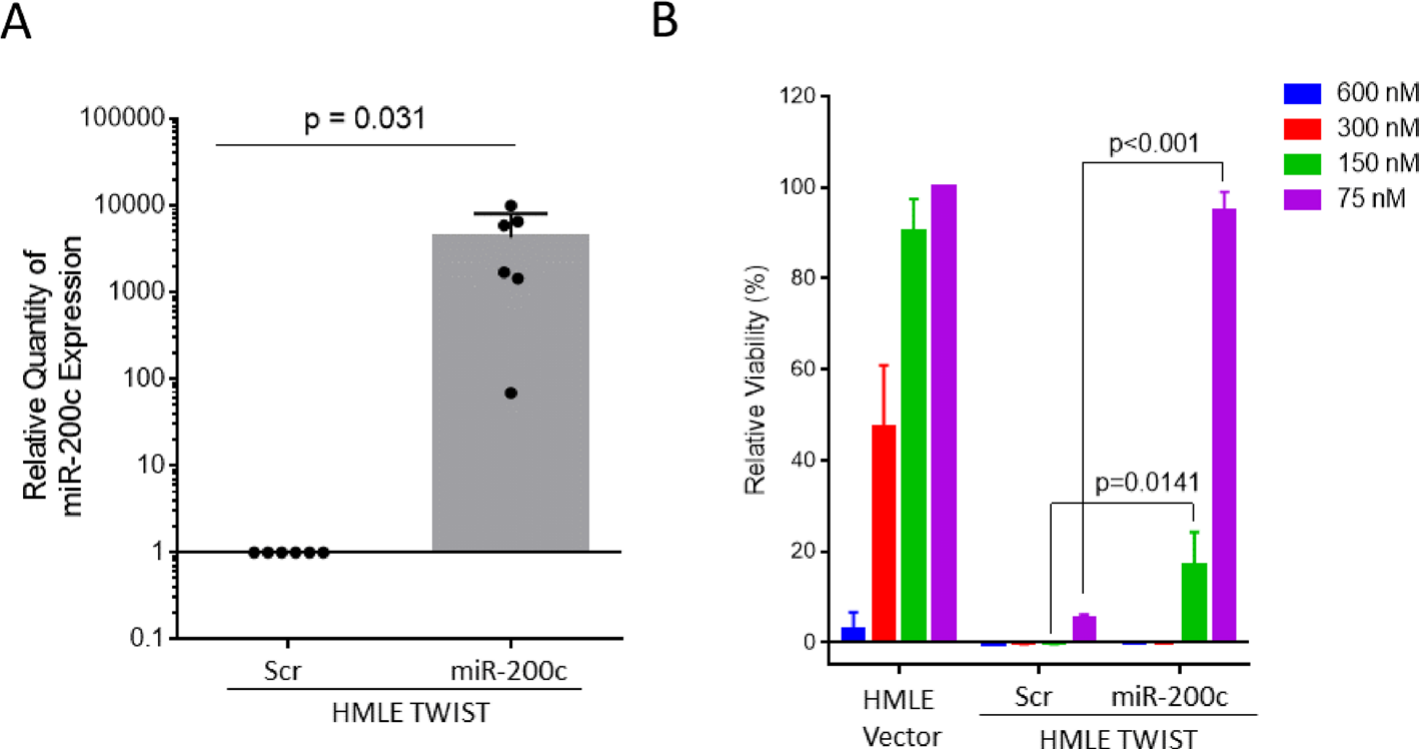
miR-200c overexpression enhances sensitivity to OpA. (**A**) Mean and standard deviation of miR-200c expression in HMLE TWIST cells expressing ectopic miR-200c or a control vector. n = 6 (**B**) Mean and standard deviation of relative viability for indicated doses of OpA in HMLE and HMLE TWIST cells expressing ectopic miR-200c or a control vector. n = 3, two-tailed student's unpaired t-test used to test significance.

Because we observed that OpA selectively impacts cells that have undergone EMT, we next evaluated whether reversing the EMT status of these cells would be sufficient to undermine OpA sensitivity. To do this, we introduced an epithelial-specific microRNA into HMLE-TWIST cells. miR-200c expression has been shown to be sufficient to reverse EMT and associated CSC features^53^. First, we verified over-expression of miR-200c in HMLE Twist cells (Fig. 2A) and confirmed the expected effects on the prevalence of CD44^hi^/CD24^lo^ cells (Sup Fig S2A) and sphere formation (Sup Fig. 2B). We next measured sensitivity to OpA and found that induction of miR-200c partially compromised sensitivity to OpA (Fig. 2B). This indicates that miR-200c-driven suppression of the CSC state impacts sensitivity to OpA.

### Persistent treatment with OpA alters cellular phenotypes

**Figure 3:**
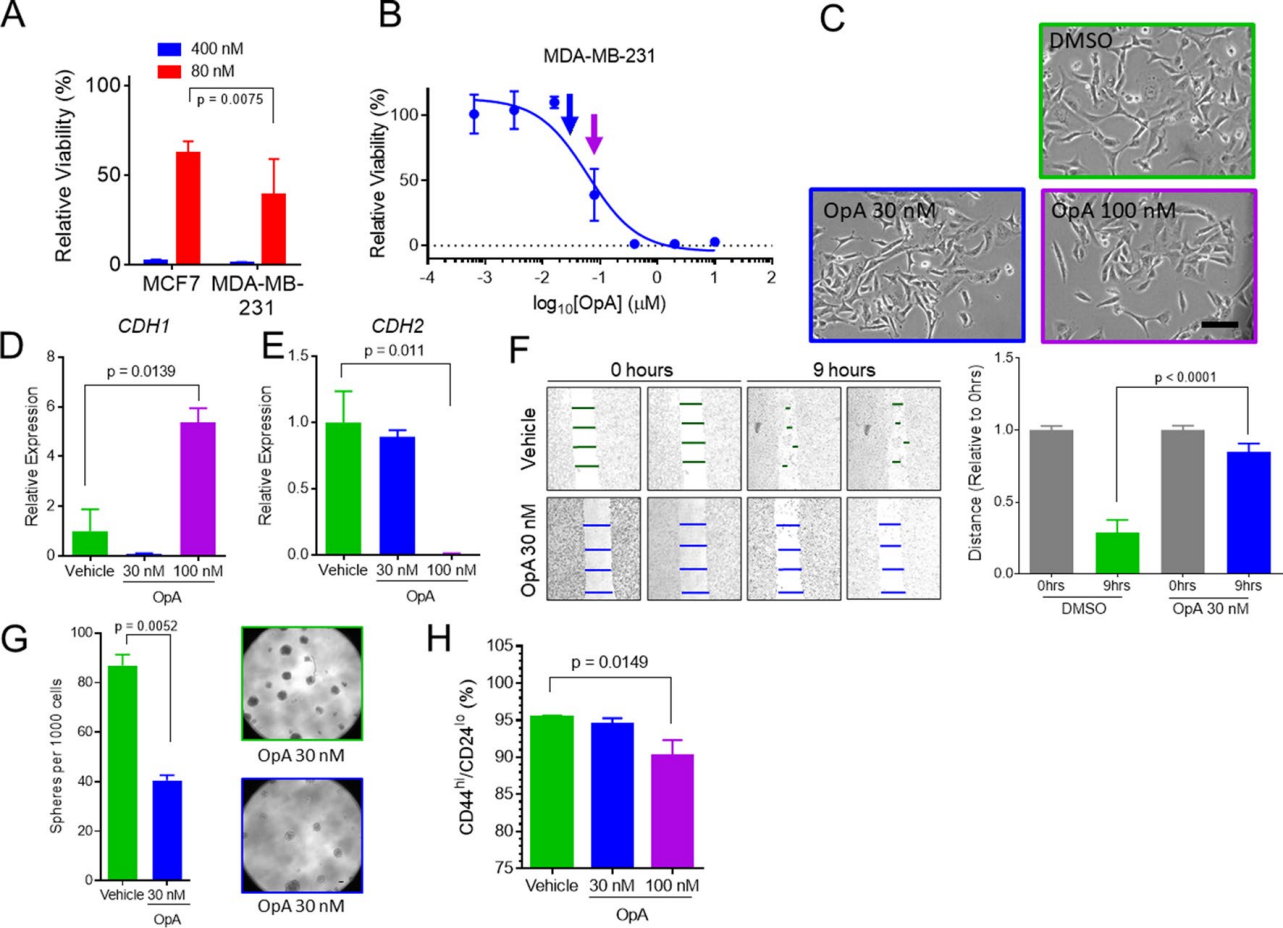
Treatment with OpA suppresses EMT-driven cell behavior. (**A**) Mean and standard deviation of relative viability for indicated doses of OpA in MCF7 and MDA-MB-231 cells, n = 8, two-tailed student's unpaired t-test used to test significance. (**B**) Representative data indicating cytotoxicity of OpA to MDA-MB-231 at the indicated doses. Blue and purple arrows indicate doses used for sub-cytotoxic pre-treatment in (**C**–**H**). (**C**) Representative morphology of MDA-MB-231 cells treated with OpA at 30 nM or 100 nM for 4 days followed by 24 h of culture in clean media. Scale bar = 100 µm. (D/E) qRT-PCR for CDH1 (**D**) and CDH2 (**E**) from cells treated as in (**C**), n = 3, mean and standard deviation are shown, two-tailed student's unpaired t-test used to test significance. (**F**) MDA-MB-231 cells, treated with OpA at 30 nM for 4 days, were cultured in clean media for 12 h then subjected to a wound healing assay. The percentage of cells with both high CD44 and low CD24 is shown. Mean and standard deviation are shown; n = 3, two-tailed student's unpaired t-test used to test significance. Representative images are shown from 0 h post-scratch and 9 h post-scratch. (**G**, **H**) MDA-MB-231 cells, treated as in (**C**) were subjected to a (**F**) sphere-forming assay n = 8, unpaired t-test, scale bar = 100 μm or a (**G**) flow cytometry assay for CD44 and CD24.

Triple-negative breast cancer cell lines of the basal-like or claudin-low subtype typically exhibit greater EMT and CSC features^54^. To examine the effect of OpA on breast cancer cells, we measured the cytotoxic activity on the ER-positive, CSC-poor, epithelial-like MCF7 and triple-negative, CSC-rich, mesenchymal-like MDA-MB-231 cell lines. While both cell lines were highly responsive to an elevated dose of OpA (400 nM), the MDA-MB-231 cells displayed significantly greater cell death at an 80 nM dose, compared to MCF7 cells (Fig. 3A). We next considered whether exposure to OpA, in addition to exerting a cytotoxic effect, might also abrogate EMT and CSC-associated cell phenotypes. To evaluate the impact of sub-cytotoxic doses of OpA on EMT and CSC phenotypes, we performed experiments on CSC-rich MDA-MB-231 cells using continuous, multi-day treatment of 30 nM OpA, 100 nM OpA, (Fig. 3B—blue and purple arrows, respectively), or vehicle. Continuous treatment with a sub-cytotoxic doses of OpA triggered modest changes in cell morphology toward a more compact and cobblestone-like appearance, characteristic of epithelial cells (Fig. 3C). To evaluate the effect on EMT, we measured markers of EMT following exposure to OpA. Cells treated with 100 nM OpA, but not 30 nM, showed increased expression of *CDH1* (E-cadherin) and decreased expression of *CDH2* (N-cadherin), indicative of a partial EMT reversion (Fig. 3D,E). As EMT is necessary for the migratory capacity of MDA-MB-231 cells, we ascertained whether OpA could inhibit migration using a wound healing assay. Consistent with an effect on EMT properties, cells pre-treated with sub-cytotoxic doses of OpA failed to migrate in response to a scratch wound (Fig. 3F). We next measured the effect of OpA on anchorage-independent growth using a mammosphere assay and on the prevalence of CSC-associated CD44^hi^/CD24^lo^ cells. Consistent with an effect on CSC properties, we observed that pre-treatment of MDA-MB-231 cells with OpA reduced sphere formation (Fig. 3G). While pre-treatment at 30 nM had no effect on the prevalence of CD44^hi^/CD24^lo^ cells, pre-treatment at 100 nM had a minor, though statistically significant effect (Fig. 3H). In summary, persistent treatment of a CSC-rich breast cancer cell line with OpA diminishes sphere formation and migratory properties associates with CSC and EMT.

### OpA treatment increases sensitivity to chemotherapy

**Figure 4:**
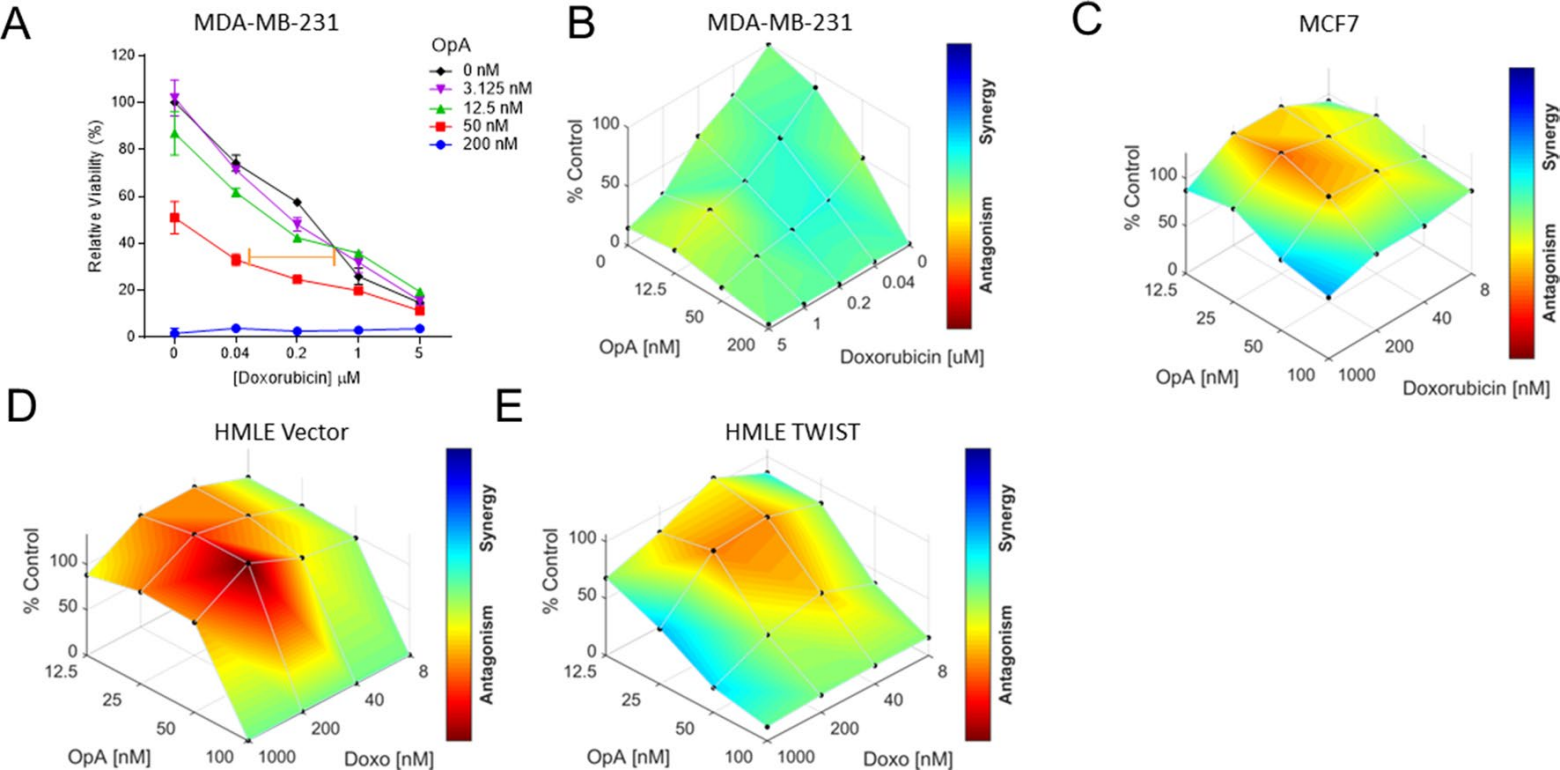
EMT-associated combinatorial activity for OpA with doxorubicin. (**A**,**B**) Representative data indicating cytotoxicity to a range of doses of OpA and doxorubicin for MDA-MB-231 (**A**), n = 4. (**B**) Data from (**A**) are represented using Combenefit. Blue-shaded areas represent dose combinations with synergistic effects. (**C**–**E**) Representative data indicating interactions between OpA and doxorubicin for MCF7 (**C**), HMLE Vector (**D**) and HMLE TWIST (**E**) cells.

EMT-promoted stemness drives resistance to commonly used chemotherapies. One approach to overcoming this problem is to consider dual-treatment therapies that combine CSC-targeting compounds with conventional drugs. To examine the relationship between EMT and the combinatorial impact of OpA treatment, we co-treated cells with a dilution series of OpA and either doxorubicin or paclitaxel. Co-treatment of MDA-MB-231 cells with as little as 12.5 nM OpA enhanced the cytotoxic response from doxorubicin (Fig. 4A), while 50 nM OpA enhanced the cytotoxic response from paclitaxel (Sup. Fig. 3A). Notably, addition of 50 nM OpA was sufficient to maintain cytotoxic activity despite a 25-fold reduction in the dose of doxorubicin (Fig. 4A-orange bar) and a fivefold reduction in the dose of paclitaxel (Sup. Fig. 3A-orange bar).When analyzed using Combenefit^55^, these dose combinations tended toward synergistic effects (Fig. 4B, Sup. Fig. 3B). Combination treatment using the synthetic derivative, 3-deoxy-OpA (Sup. Fig. 3C) did not result in altered activity (Sup. Fig. 3D–F). To test whether the EMT state or degree of CSC enrichment was relevant for synergistic activity of OpA and doxorubicin, we performed combination treatment analysis on MCF7, HMLE vector and HMLE TWIST cells. MCF7 cells displayed less synergy and more antagonism than MDA-MB-231 cells (Fig. 4C). HMLE vector cells treated with OpA and doxorubicin also exhibited strong antagonism which was diminished in the HMLE TWIST cells (Fig. 4D,E). The capacity of OpA to act in concert with clinically useful chemotherapeutic agents indicates that co-treatment may be useful to more effectively treat breast cancer.

### OpA is tolerated in vivo and suppresses growth of mammary cell tumors with exogenous TWIST-expression

**Figure 5:**
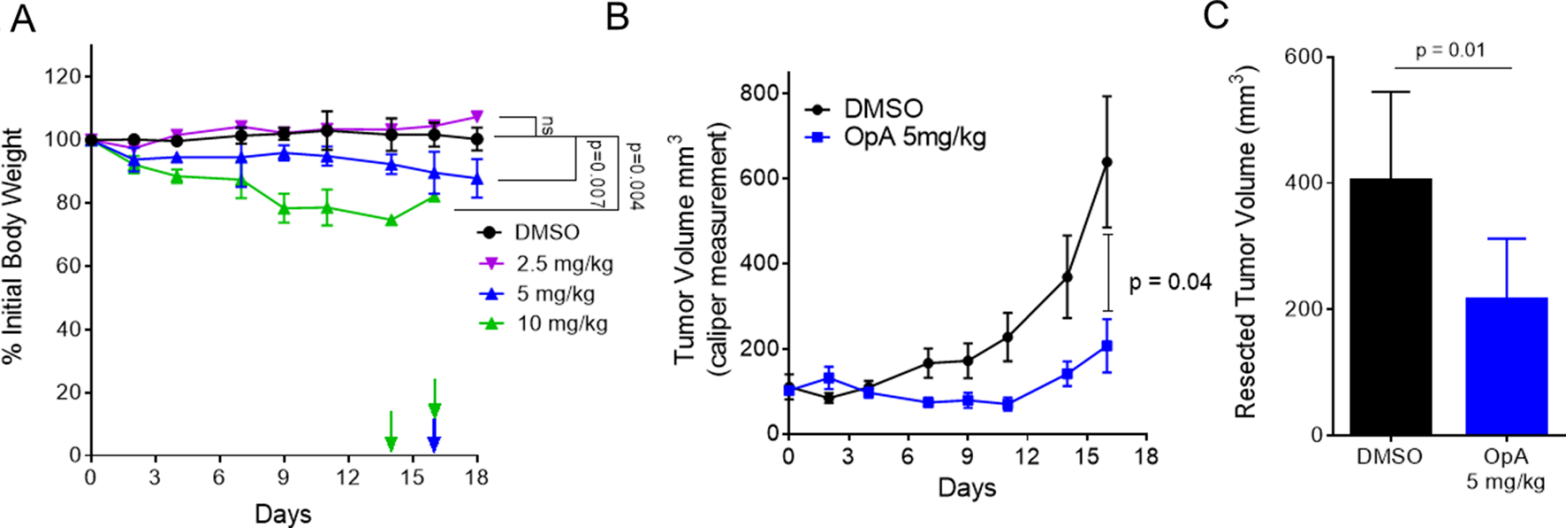
OpA is tolerated in vivo and suppresses tumor growth from cells over-expressing TWIST. (**A**,**B**) Mice, bearing tumors composed of HMLER-TWIST cells, were injected with 10 mg/kg (n = 2), 5 mg/kg (n = 5), 2.5 mg/kg (n = 3) of OpA, or vehicle control (n =5), thrice weekly for three weeks. (**A**) Body weight was tracked. Arrows indicate endpoint criterion met for an individual animal. Statistical significance measured using the Holm-Sidak method with an alpha of 5%. (**B**) Tumor volume was measured at the indicated timepoints by caliper and is given as length × width^2^ divided by 2. n = 5. Statistical significance of the difference between volumes at 17 days is indicated as determined by two-tailed student's unpaired t-test. (**C**) End-point tumor volume was compared by two-tailed student's unpaired t-test, n = 4.

We next assessed whether OpA treatment alone is sufficient to reduce growth of a mammary cell tumor, which is composed exclusively of EMT-positive cells, in mice. As such, immunocompromised mice were orthotopically injected with Ras-transformed HMLE cells constitutively expressing the TWIST transcription factor to induce EMT (HMLER-TWIST). Following the emergence of palpable tumors, mice were randomly assigned to either the control (DMSO diluted into saline) or OpA treatment groups. Thrice weekly injections for 3 weeks consisting of 10 mg/kg of OpA were not well tolerated as mice exhibited weight loss greater than 20% of initial body weight and two adverse outcomes were recorded prior to the final dose (Fig. 5A). However, a dose of 5 mg/kg was better tolerated with weight loss less than 15% and one adverse outcome, while a dose of 2.5 mg/kg had no statistically significant impact on body weight (Fig. 5A). A dose of 5 mg/kg of OpA was sufficient to significantly suppress the growth of HMLER-TWIST tumors (Fig. 5B) and to reduce the endpoint tumor volume of HMLER-TWIST tumors (Fig. 5C). We sought to ascertain whether OpA treatment contributes to increased cell death within treated tumor tissue by staining for cleaved caspase-3, a marker of apoptosis. Unexpectedly, staining for cleaved caspase-3 in the primary tumors revealed no significant difference between untreated and OpA-treated mice (Sup. Fig. 4). However, OpA has been shown to induce non-apoptotic cell death in other models^39,56^. Because HMLER-TWIST tumors metastasize to the lung and other organs^25^, we analyzed lungs from OpA-treated mice to determine if metastatic burden was reduced. Despite the observed effects on migration in vitro, there was no significant reduction in lung metastatic burden associated with OpA treatment (Sup. Fig. 4).

## Discussion

Currently, conventional chemotherapeutic drugs are able to elicit high response rates in about half of TNBC patients; however, the remaining patients eventually develop progressive disease^2^, with some even experiencing more aggressive and CSC-rich tumors after therapy^14,57^. Identification of molecules with specificity for CSC-rich cell populations will facilitate the development of novel therapies and may improve responses to currently available therapies.

While several other natural products have been linked to CSC-targeting^33,34,48,58–63^, our work highlights a natural product that selectively kills breast CSCs exhibiting EMT features. Further, we show a reduction of EMT phenotypes such as migration, as well as reduction in sphere-forming capacity and changes to CSC-rich subpopulations in a TNBC cell model. Extending OpA’s efficacy in reducing CSC-related properties, our data suggest increased sensitivity to conventional chemotherapeutics doxorubicin and paclitaxel when co-treated with OpA. Finally, we evaluate the efficacy of OpA in vivo and show suppressed growth of an EMT-positive, primary tumor.

Evolution-driven selection of natural products imparts biological activities useful for disease treatment and which may not be mimicked by selective kinase inhibitors. Other successful natural products that have driven cancer therapies include taxol, vinblastin, anthracyclines, daunomycin and doxorubicin^64^. Several studies^39,41,42,46,65–68^ have evaluated one such natural product, OpA, in cancer settings, predominantly using in vitro models, and, similar to our present study, these studies report IC_50_ values in the low nanomolar range. Our work is one of the first to evaluate OpA in vivo and is the first to describe the impact of EMT on OpA sensitivity. By focusing on the effects on EMT and stemness phenotypes, this work opens the door for the discovery of essential molecular pathways and for the investigation of OpA derivatives as a future cancer treatment.

## Materials and methods

### Cell lines

MCF7, and MDA-MB-231, were received from ATCC; HMLE, HMLER, HMLE Snail, HMLER Snail, HMLE TWIST, and HMLER TWIST were kindly gifted from Dr. Sendurai Mani (MD Anderson Cancer Center). Breast cancer cells were cultured in Dulbecco’s Modified Eagle’s Medium (DMEM) (Corning Inc., Kennebuck, ME, USA) supplemented with 10% fetal bovine serum (FBS) (Equitech-Bio Inc., Kerrville, Texas, USA) and 1X antibiotics (Penicillin/Streptomycin, Lonza, Basel, Switzerland). Immortalized human mammary epithelial cells (HMLE) and derivatives were maintained as in Elenbaas et al.^69^. Cell lines were tested monthly for mycoplasma and validated via STR testing. Incubation occurred at 37 °C with 5% CO_2_. miR-200c overexpression was generated using lentiviral transduction of pCMV-MIR (Origene Rockville, MD). Transduced cells were selected using puromycin.

### Reagents

Curcumin, genestein, doxorubicin, and paclitaxel were obtained from Selleckchem (Houston, TX, USA), salinomycin from Cayman Chemicals (Ann Arbor, MI USA), and disulfiram from Tocris Bioscience, (Bristol, UK). OpA was produced by fermentation of the fungus *D. gigantea*. It was extracted from the fungal culture filtrates, purified, crystallized and identified by ^1^H NMR and ESI MS spectra as previously reported^70^. The purity of OpA was > 98% as ascertained by ^1^H NMR and HPLC analyses.

3-Deoxy OpA was synthesized from ophiobolin I^71,72^ which was also obtained through fermentation as previously reported^70^. A two-step synthetic sequence involving conjugate reduction of the enone which proceeded with high diastereoselectivity (> 19:1 by 600 MHz ^1^H NMR) followed by a Ru(IV)-mediated oxidation of the primary alcohol to the aldehyde delivered 3-deoxy OpA. It should be noted that the methyl group at C3 is epimeric with respect to the C3-methyl group in OpA. However, the importance of the C3-hydroxy group and/or the stereochemistry of this methyl group was verified through studies described below and 3-deoxy OpA served as a negative control. Further details are provided in Supplemental Figure 5.

### Viability

Cells were plated with 2000 cells per well in a 96-well plate and allowed to adhere overnight. Compounds, suspended in DMSO and diluted into PBS, or vehicle were added to the culture medium and incubated for 72 h at 37 °C, 5% CO_2_. Following manufacturer suggested protocol, 20 µL CellTiter 96^®^ AQ_ueous_ One Solution Cell Proliferation Assay (MTS; Promega, Madison, WI, USA) was added and incubated 1–4 h at 37 °C, 5% CO_2_. Absorbance was measured at 490 nm using a 96-well plate reader (Fisher Scientific, Hampton, NH, USA).

### RNA extraction and detection

Cells were lysed in the presence of Trizol^®^ Reagent (Thermo Scientific, Waltham, MA, USA) and total RNA extracted following manufacturer protocol recommendations. Relative quantification of the mRNA levels was performed using the comparative Ct method with the formula 2^−ΔΔCt^. For microRNA analysis small nucleolar RNA U6 was used for normalization while for mRNA analysis GAPDH was used for normalization, Taqman and SYBR PCR Master Mixes were obtained from Applied Biosystems (Thermo Scientific, Foster City, CA, USA). All quantitative reverse transcription-PCR (RT-PCR) experiments were run in technical quadruplicates and biological triplicates and a mean value was used for the determination of mRNA levels.

### Western blotting and antibodies

Cells were lysed in the presence of 100 µl radio-immunoprecipitation (RIPA) buffer containing protease inhibitors (Alfa Aesar, Stoughton, MA, USA) on ice. Protein was quantified using the Bradford Assay (BioRad, Hercules, CA, USA). Twenty micrograms of total protein from each sample was resolved on a 4–12% SDS-PAGE gel and transferred to PVDF membranes. Sister blots were then probed with antibodies including anti-E-cadherin (Cell Signaling, Danvers, MA, USA), anti-vimentin (Protein Technologies, Tucson, AZ, USA), or anti-β-actin (BD Biosciences, San Jose, CA) antibody. Chemiluminescent signals were detected with ECL™ prime (Thermo Fisher Scientific) using the Biorad ChemiDoc system. If necessary, blots were stripped with ECL Stripping Buffer (Li-Cor, Lincoln, NB, USA) following manufacturer protocol. Bands were quantified using ImageJ.

### Mammosphere assay

Cells were harvested and suspended in serum-free mammary epithelial growth medium (MEGM) supplemented with 1% methyl cellulose, 20 ng/mL FGF, 10 ng/mL EGF, and 4 μg/mL heparin. Cells were plated in 4 replicates in a flat-bottom ultra-low attachment 96-well plate (Corning) and allowed to grow at 37 °C, 5% CO_2_ for 10–14 days and were monitored microscopically to ensure that they did not become confluent during the experiment. 100 µL low-attachment media was added every 3–4 days. Wells were imaged using 4× magnification on a computer-assisted phase contrast microscope (Nikon, Tokyo, Japan). Spheres larger than 100 µm were counted.

### Flow cytometry

For flow cytometry, cells were harvested, counted and 10^5^ cells were incubated with 5 µl of either CD44 (BV421 Mouse Anti-Human CD44 # 562890; BD Biosciences, San Jose, CA, USA) and/or CD24 (PE-Mouse Anti-human CD24 #555428; BD Biosciences) in PBS with 1% serum for 1 h on ice, minimizing light exposure. Cells were then pelleted at low-speed and washed with PBS with 1% serum twice before measurement of fluorescence using BD FACS Melody (BD Biosciences).

### Migration

For migration assay, cells were serum-starved overnight and scratch wounds were created using a sterile pipette tip on the cell monolayer or by plating cells in 2-well culture inserts (Ibidi, Madison, WI). Cell migration rates were determined by measuring the distance between cell fronts after the indicated number of days in culture. The distance between the two edges at multiple points was quantified using ImageJ at the indicated timepoints.

### Co-treatment and interaction

Cells were treated with compound or matched-percentage DMSO or other vehicle in serial dilutions and incubated for 72 h before measuring viability using MTS (Promega, Madison, WI, USA). Interactions were quantified using the Combenefit program with the Loewe model and dose–response surface mapping^55^.

### Tumor growth

Female Scid/bg (CB17.Cg-PrkdcscidLystbg-J/Crl) mice (5–8 weeks old) were obtained from Charles River Laboratories (Wilmington, MA, USA)**.** Animals were maintained under clean room conditions in sterile filter top cages with autoclaved bedding and housed on high efficiency particulate air–filtered ventilated racks. Animals received sterile rodent chow and acidified water ad libitum. All of the procedures were conducted in accordance with the Institute for Laboratory Animal Research Guide for the Care and Use of Laboratory Animals and with Baylor University Animal Care and Use Committee guidelines. HMLER-TWIST cells were harvested, pelleted by centrifugation at 2000×*g* for 2 min, and resuspended in sterile serum-free medium supplemented with 30% to 50% Matrigel (BD Biosciences, San Jose, CA, USA). Cells (2 × 10^6^ in 100 µl aliquots) were implanted into the left fourth mammary fat of each mouse and allowed to grow until measurable by caliper. Then, OpA or vehicle was administered by intraperitoneal injection three times weekly for 3 weeks at 2.5 mg/kg, 5 mg/kg or 10 mg/kg. Tumor volume and body weight were recorded concurrently with injection protocol^73^. At designated times, mice were humanely euthanized, and tumors and lungs were collected. Experiments were approved by Baylor University IACUC (#1441130). This study was carried out in compliance with ARRIVE guidelines (http://www.nc3rs.org.uk/page.asp?id=1357).

### Tissue staining

Immunohistochemistry was performed on formalin-fixed, paraffin-embedded tissue. The Leica Bond Max automated platform was used to perform the immunohistochemistry. The antibodies used were as follows: Caspase 3 Lot#GR3265151-4 (Abcam, Cambridge, MA). Antibodies were diluted at 1:1000.

### Statistical analysis

Unless otherwise stated, statistical differences were determined using a student’s t-test. The GraphPad PRISM software v6 was used to perform these analyses. Statistical significance levels are annotated as n.s. = non-significant, *p < 0.05, **p < 0.01, ***p < 0.001, ****p < 0.0001.

## Supplementary Information

Supplementary Information.
