# Supplementary material for: Epithelial-mesenchymal transition sensitizes breast cancer cells to cell death via the fungus-derived sesterterpenoid ophiobolin A
Source: Sci Rep. 2021 May 20;11:10652. doi: 10.1038/s41598-021-89923-9 (PMC8137940; doi:10.1038/s41598-021-89923-9)
Supplement: Supplementary file 1 — Supplementary Information. [file 41598_2021_89923_MOESM1_ESM.pdf]

Epithelial-mesenchymal transition sensitizes breast cancer cells to cell death via the fungus-derived sesterterpenoid ophiobolin A

Keighley N. Reisenauer[1], Yongfeng Tao[2], Provas Das[1], Shuxuan Song[1], Haleigh Svatek[1], Saawan D. Patel[1], Sheridan Mikhail[1], Alec Ingros[1], Peter Sheesley[1], Marco Masi[3], Angela Boari[4], Antonio Evidente[3], Alexander V. Kornienko[5], Daniel Romo[2], Joseph Taube[1#]

Affiliations

[1]Department of Biology, Baylor University, Waco, TX, USA

[2]Department of Chemistry and Biochemistry, Baylor University, Waco, TX, USA

[3]Department of Chemical Sciences, University of Naples Federico II, Complesso Universitario Monte Sant'Angelo, Naples, Italy

[4]Institute of Sciences and Food Production, CNR, Bari, Italy

[5]Department of Chemistry and Biochemistry, Texas State University, San Marcos, TX, USA

[#]Corresponding author: Joseph Taube, Joseph_Taube@baylor.edu

Supplemental Data

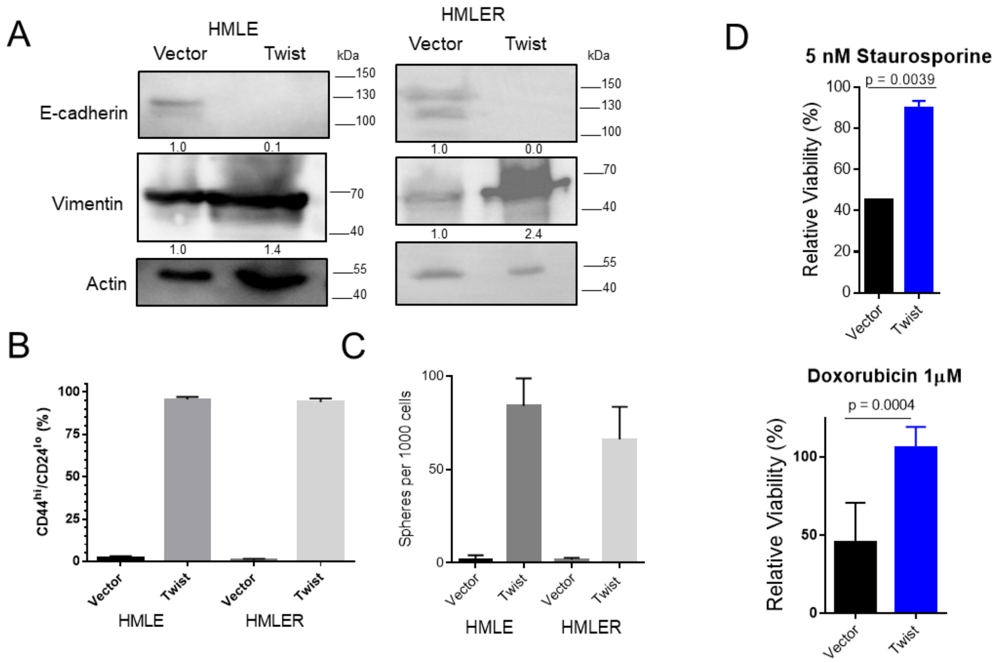

Supplemental Figure 1: EMT and CSC features of cells overexpressing Twist.

(A) Representative western blot showing E-cadherin and vimentin protein expression. Images cropped to show relevant bands. Band intensity is normalized to actin and provided as a quantification relative to vector cells. Full-length blots available. (B) Flow cytometry was performed to measure expression of CD44 and CD24. The percentage of cells with both high CD44 and low CD24 is shown. Mean and standard deviation are shown, n = 3. (C) Mammosphere formation assay was performed on the indicated cells. Mean and standard deviation are shown, n = 4. (D) HMLE cells with the indicated vectors were exposed to the indicated dose of the indicated compounds for 72 hours before viability was assessed using an MTS assay. Data are normalized to vehicle treatment. Mean and standard deviation are shown, n = 4. Students t-test used to generate p-values.

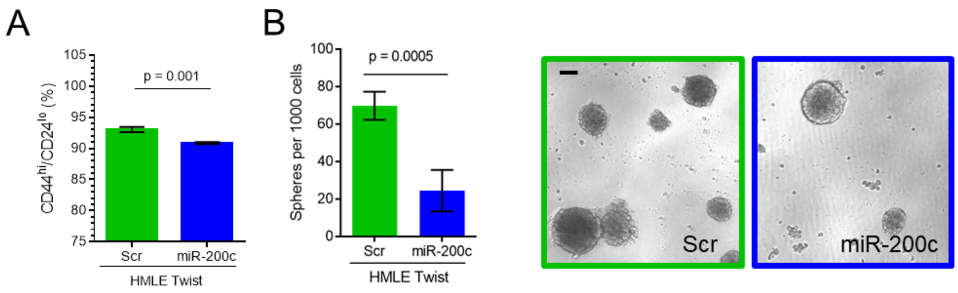

Supplemental Figure 2: Suppression of CSC features of cells overexpressing miR-200c.

(A) Flow cytometry was performed to measure expression of CD44 and CD24. The percentage of cells with both high CD44 and low CD24 is shown. Mean and standard deviation are shown, n = 3. (B) Mammosphere formation assay was performed on the indicated cells. Representative images are included, scale bar = 100 µm. Mean and standard deviation are shown, n = 4. Students t-test used to generate p-values.

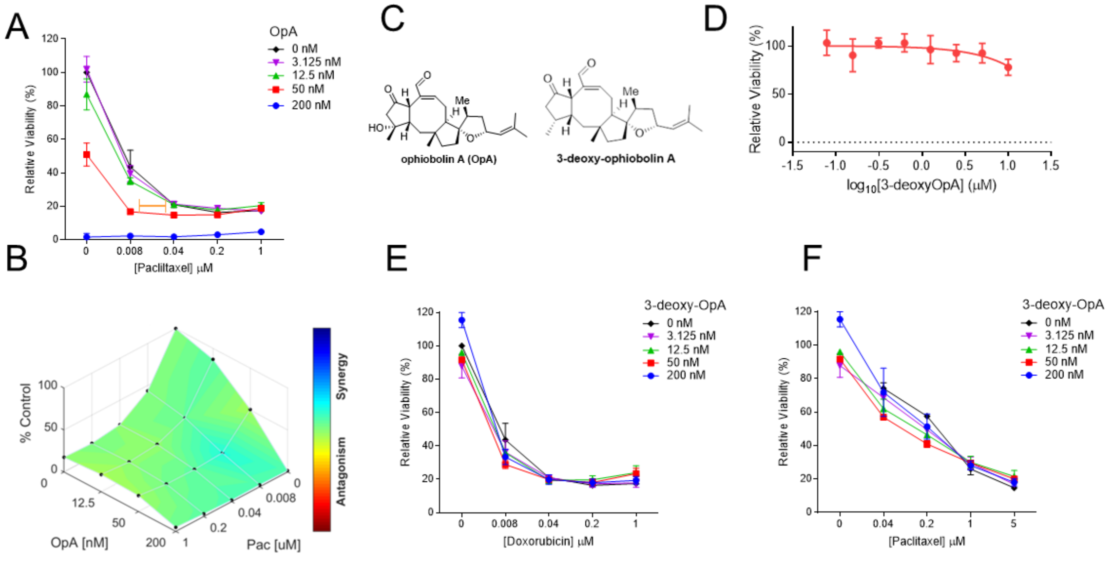

Supplemental Figure 3: OpA synergizes with paclitaxel while deoxy-OpA is inactive towards MDA-MB-231 cells

(A) Representative data indicating cytotoxicity to a range of doses of OpA and paclitaxel for MDA-MB-231. (B) Data from (A) are represented using Combenefit. Blue-shaded areas represent dose combinations with synergistic effects. (C) Molecular structures of OpA and deoxy-OpA. (D) Representative data indicating cytotoxicity of deoxy-OpA to MDA-MB-231 at the indicated doses. (E/F) Representative data indicating cytotoxicity of MDA-MB-231 to a range of doses for 3-deoxy-OpA and doxorubicin (E) or 3-deoxy-OpA and paclitaxel (F).

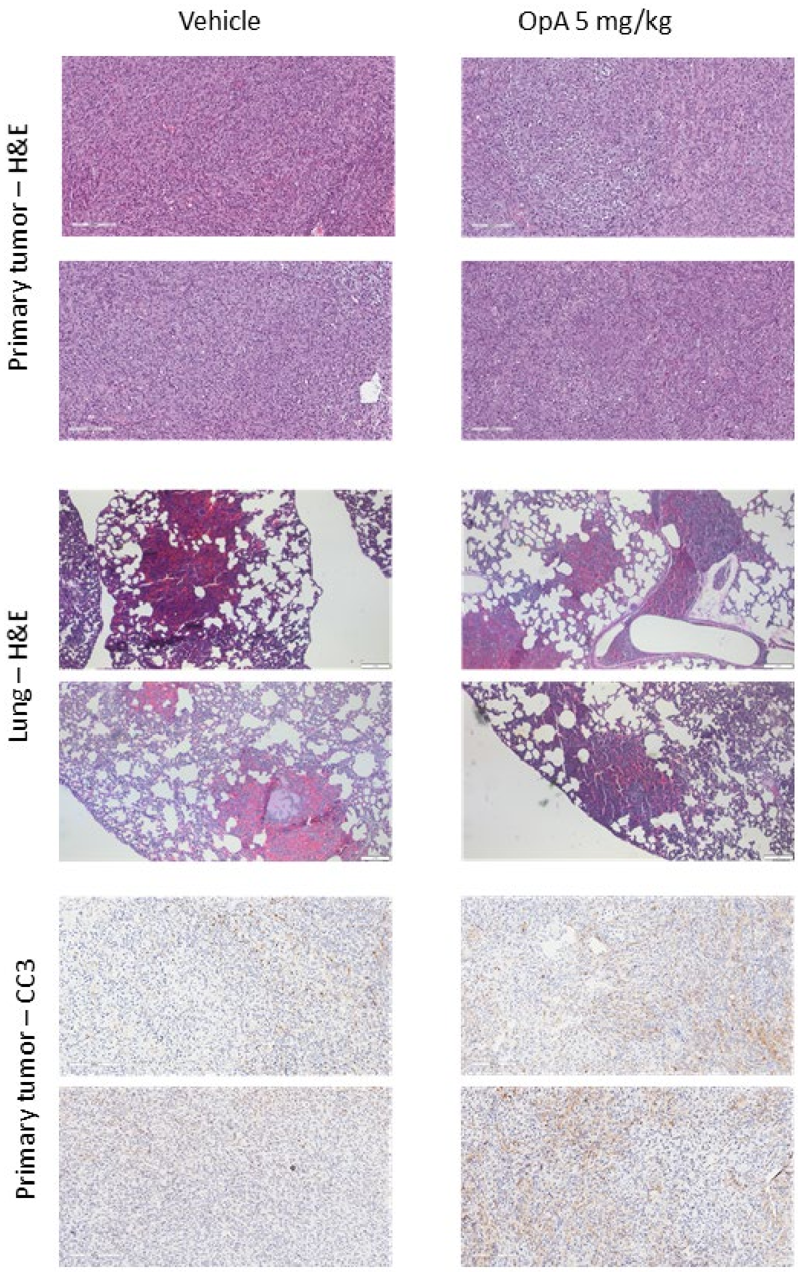

Supplemental Figure 4:

Histology of primary tumors and lungs of mice xenografted with HMLER Twist cells and treated with OpA or vehicle.

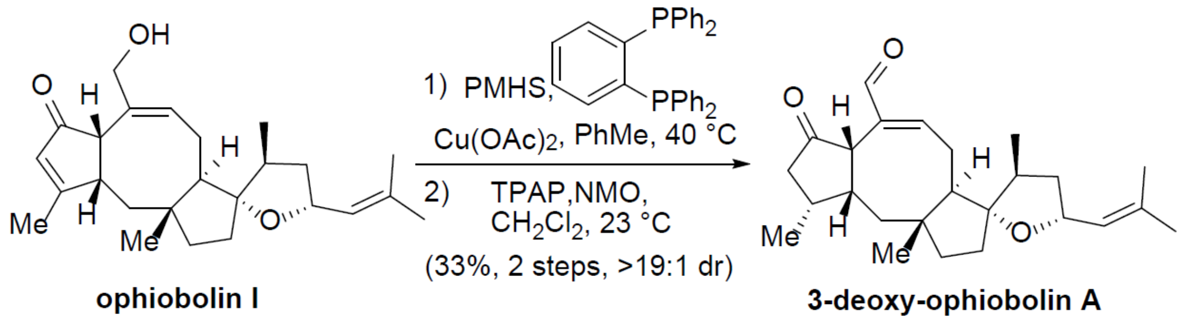

Supplemental Figure 5: Synthesis of 3-deoxy-ophiobolin A.

To a dry 1-dram vial was added 3 mL dry PhMe, Cu(OAc)2•H2O (13 mg, 0.065 mmol, 5 equiv), 1,2-bis(diphenylphosphino)benzene (3.0 mg, 0.007 mmol, 0.5 equiv), t-BuOH (0.18 mL, 1.95 mmol, 150 equiv), polymethyl hydrosilane (PMHS) (0.12 mL, 1.95 mmol, 150 equiv). The mixture was stirred at 23 oC under argon for 30 min to generate a stock solution of the required reducing agent. In another dry 1-dram vial, ophiobolin I1 (3.0 mg, 0.008 mmol, 1 equiv) was dissolved in 0.5 mL dry PhMe, and 0.3 mL of the above reagent stock solution was added. The reaction was heated to 40 oC under argon and stirred at this temperature for 2 h. The mixture was concentrated and filtered through a pad of silica gel with CH2Cl2 as eluent. The filtrate was then concentrated, dissolved in CH2Cl2, and then N-methyl morpholine N-oxide (NMO, 1.2 mg, 0.01 mmol, 2 equiv) and N-tetra n-propyl ruthenium tetroxide (TPAP, 0.1 mg, 0.0003 mmol, 0.05 equiv) were added and stirred at 23 oC for 1 h. The mixture was concentrated and analysis of the crude 1H NMR (600 MHz) suggested formation of a single diastereomer during the conjugate reduction (>19:1). The mixture was then purified by silica gel chromatography (0 → 10% EtOAc/hexanes) to afford 3-deoxy ophiobolin A (1.0 mg, 33%, >19:1 dr by 1H NMR) as a colorless residue: TLC (EtOAc: hexanes, 1:2 v/v): Rf = 0.3; 1H NMR (600 MHz, CDCl3) δ 9.15 (s, 1H), 6.78 (dd, J = 7.0, 2.4 Hz, 1H), 5.06 (dq, J = 9.2, 1.5 Hz, 1H), 4.54 (dt, J = 8.6, 7.1 Hz, 1H), 3.08 (d, J = 9.4 Hz, 1H), 2.72 (dt, J = 19.8, 3.1 Hz, 1H), 2.53 (dd, J = 14.2, 4.2 Hz, 1H), 2.48 (dd, J = 17.3, 12.6 Hz, 1H), 2.36 (ddd, J = 17.3, 6.6, 1.6 Hz, 1H), 2.30 – 2.22 (m, 1H), 2.14 (q, J = 6.9 Hz, 1H), 1.91 (dd, J = 13.6, 3.5 Hz, 1H), 1.76 – 1.67 (m, 7H), 1.64 (d, J = 1.4 Hz, 3H), 1.60 (d, J = 1.3 Hz, 3H), 1.36 – 1.25 (m, 2H), 1.09 (d, J = 5.8 Hz, 3H), 0.97 (d, J = 6.9 Hz, 3H), 0.76 (s, 3H); 13C NMR (150 MHz, CDCl3) δ 218.81, 193.72, 157.07, 142.60, 135.31, 126.74, 96.06, 71.99, 53.73, 50.81, 50.06, 47.88, 47.16, 42.27, 42.22, 42.02, 38.41, 35.33, 30.93, 29.57, 25.96, 22.73, 18.84, 18.22, 16.22; IR (thin film): 2357, 1738, 1664cm-1 ; HRMS (ESI+) m/z calcd for C25H36O3Na [M+Na]+: 407.2557, found: 407.2563.

1Ophiobolin I was kindly provided by Prof. Antonio Evidente (University of Naples, Italy).

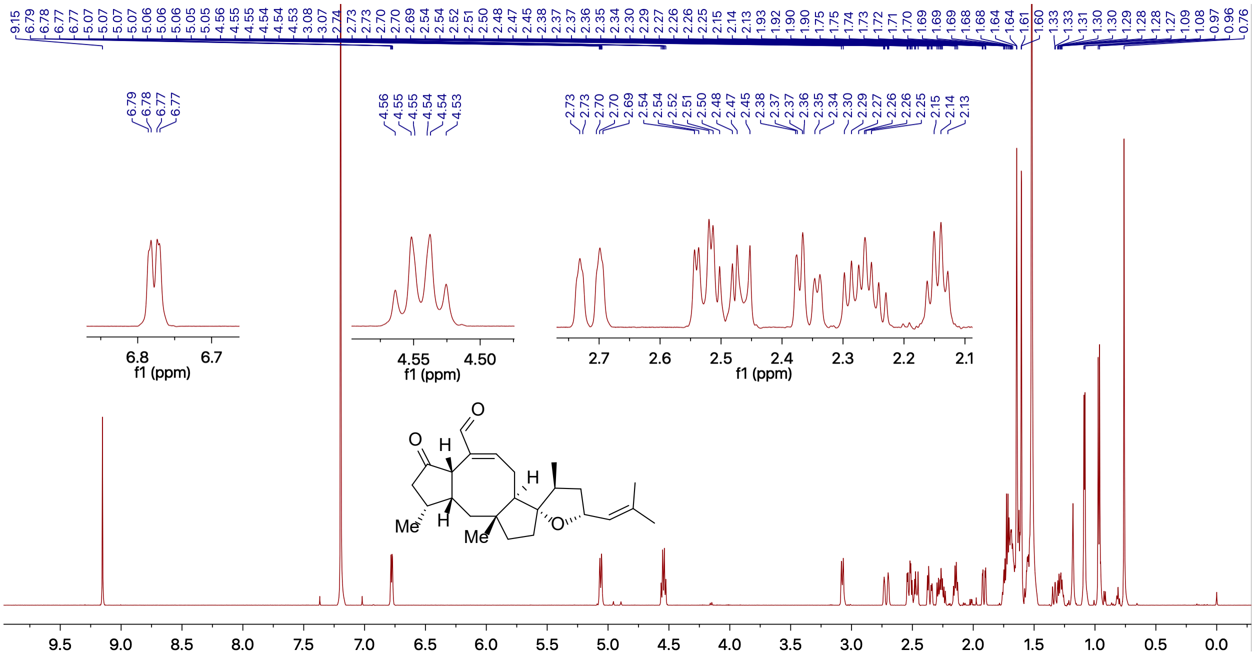

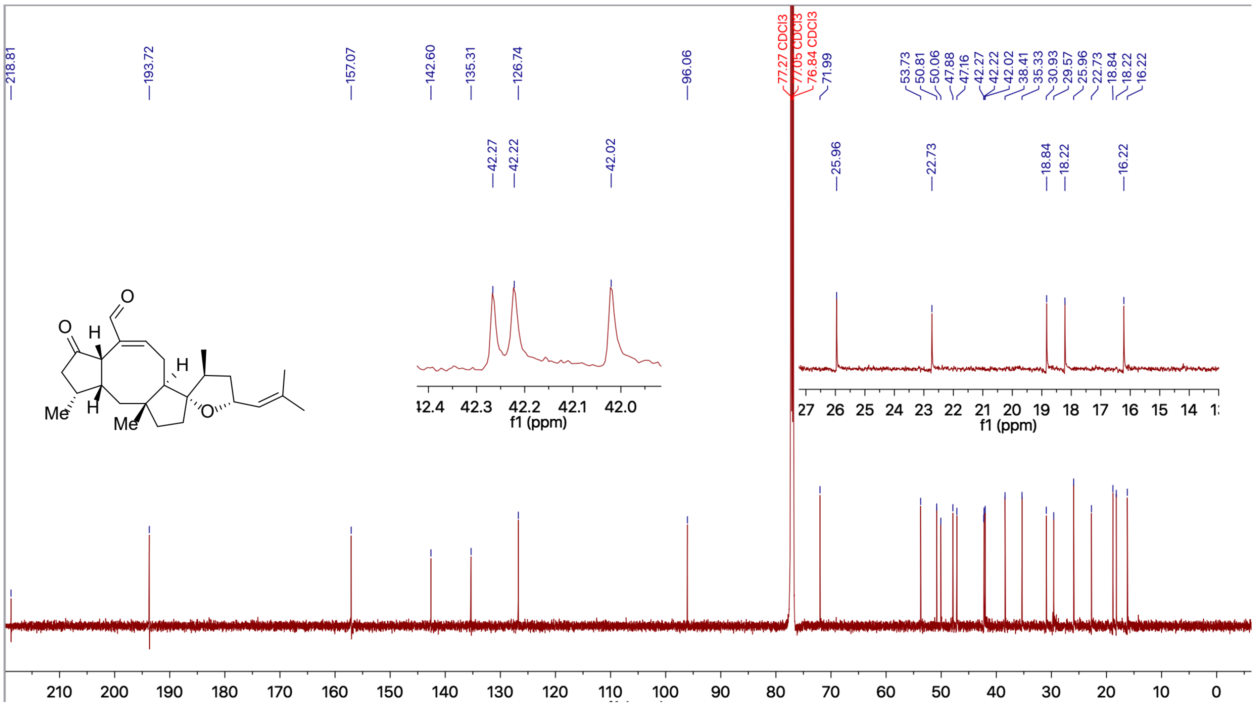

^{1}H NMR (600 MHz, $CDCl_3$) and ^{13}C NMR (150 MHz, $CDCl_3$) of **3-deoxy-OpA**

Supporting Information

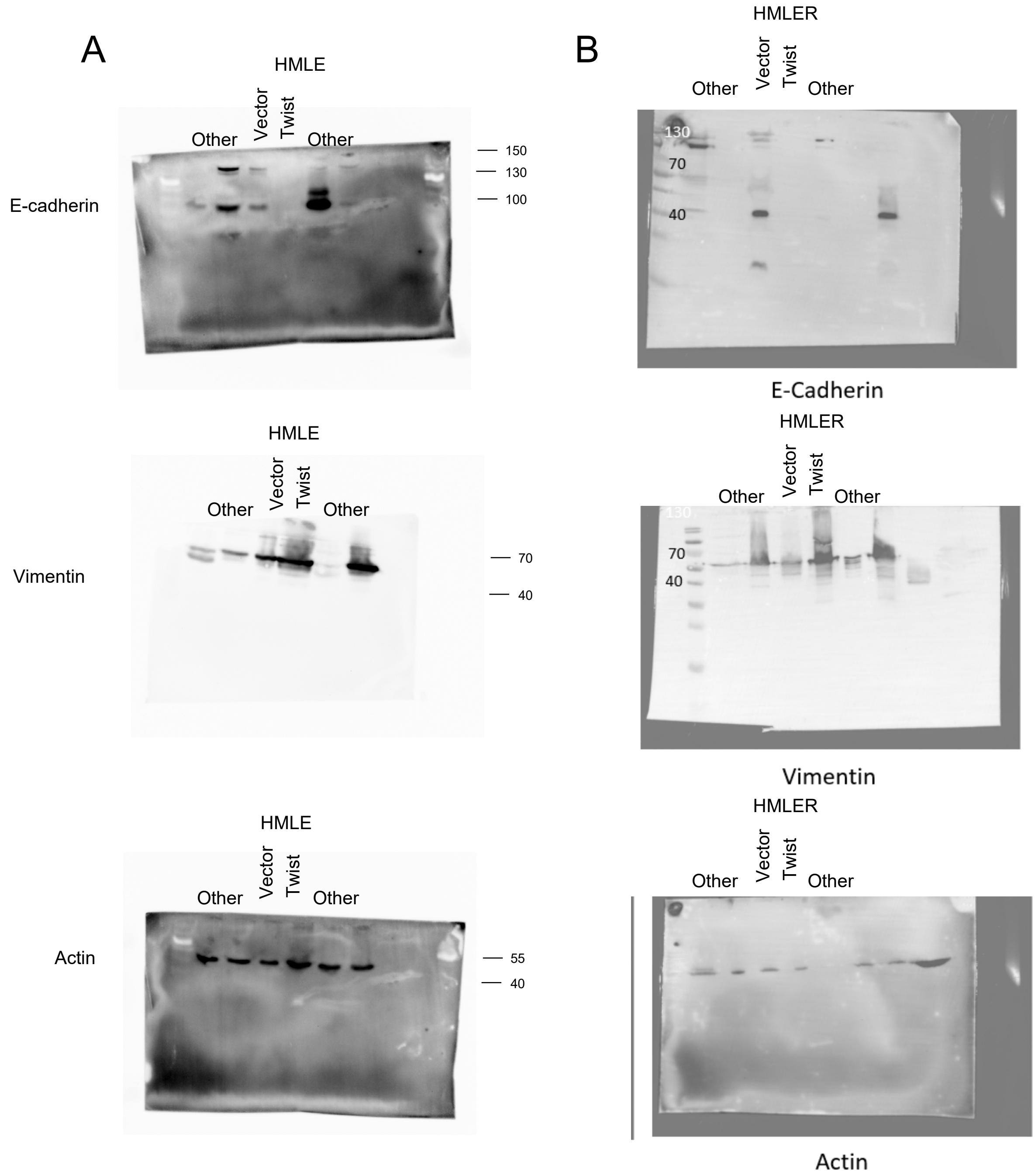

Twist-induced protein expression. Western blotting for the indicated proteins was performed on whole cell lysates from vector or Twist-transduced HMLE (A) or HMLER (B) cells. Chemiluminescence was detected and imaged. Molecular weight of ladder proteins, indicated on the right, was used to trim the membranes prior to horizontal membrane cutting for (B).
